# Effects of Çemen pastes prepared in different formulations on physicochemical, microbiological, and textural properties of beef hamburger patties during refrigerated storage

**DOI:** 10.1002/fsn3.4099

**Published:** 2024-03-15

**Authors:** Ömer Köker, Birol Kılıç, Azim Şimşek

**Affiliations:** ^1^ Faculty of Engineering, Department of Food Engineering Suleyman Demirel University Isparta Turkey; ^2^ Department of Food Processing Egirdir Vocational School Isparta University of Applied Sciences Isparta Turkey

**Keywords:** beef hamburger, çemen paste, fenugreek seed flour, garlic powder, quality, sweet red pepper

## Abstract

This study aimed to investigate the effects of çemen pastes prepared in different formulations on physicochemical, microbiological, and textural properties of hamburgers during refrigerated storage (4°C; 60 d). Çemen pastes were produced by using different combination doses of fenugreek seed flour, sweet red pepper, and garlic powder. As a result of çemen paste usage in hamburgers, cooking losses and dimensional shrinkage decreased, whereas moisture and fat retention ratios increased (*p* < .05). The hardness, gumminess, and chewiness values of hamburgers containing çemen paste were generally lower than those of control (*p* < .05). Çemen paste addition to hamburgers generally did not cause a difference in terms of microbial growth and moisture, fat and ash contents. Protein contents of hamburgers containing çemen paste or breadcrumbs were generally higher than that of control (*p* < .05). Çemen paste usage in hamburgers generally decreased the L* values and increased the b* values (*p* < .05). In general, addition of 3.5% or higher doses of both sweet red pepper and garlic powder caused higher a* values in hamburger patties (*p* < .05). Lower oxidation levels were generally observed in hamburgers containing 3% or lower doses of fenugreek seed flour and 4.5% of garlic powder in çemen paste (*p* < .05). It was concluded that çemen paste usage in hamburger patty processing has the potential to improve the quality characteristics and delay oxidative changes.

## Graphical abstract

The use of çemen paste prepared with different formulations in beef hamburgers was evaluated. Cooking and textural characteristics were improved with çemen paste addition to hamburgers. Çemen paste usage in hamburgers has the potential to improve quality features and delay oxidative changes.

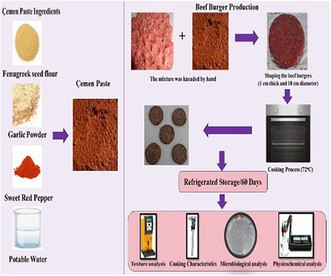

## 1 INTRODUCTION

Meat products such as hamburgers are among the most popular processed products consumed by consumers (Delgado‐Ospina et al., 2022). Hamburgers are commonly consumed as fastfoods in many parts of the world. Hamburgers are products consisting of beef meat, fat, and certain amounts of water, fillers, flour, and spices (Mancini et al., 2020). Although it is a meal with a high‐caloric value due to its high saturated fat content, hamburgers can be manufactured with quality raw materials or new formulations can be created with components that can provide some functional properties. New meat products are manufactured to satisfy consumers' demands regarding taste, appearance, value, and comfort (de Araújo et al., 2022). Many herbs, plants, fruits, and vegetable extracts or powders can be used in meat products for this purpose (Delgado‐Ospina et al., 2022). In this respect, çemen paste, which is produced and consumed locally in Türkiye, is prominent in the meat products sector.

Çemen is a paste‐like material produced by mixing water with fenugreek (*Trigonella foenum‐graecum*) seed flour, garlic, and sweet red pepper in certain proportions and used as an edible coating material in the production of pastırma, a traditional meat product. Çemen is also consumed as paste in breakfast. Recently, it has also been used in meat products, such as sausages and meatballs (Kök & Çiçek, 2022). Çemen paste formulation traditionally consists of 50% fenugreek seed flour, 35% garlic, and 15% sweet red pepper (Aksu et al., 2021). Çemen paste as a coating material contributes to creating unique taste, aroma, color, and flavor in pastırma. Furthermore, pastırma is protected from external factors owing to the çemen paste and excessive drying is prevented. Additionally, microbial growth and oxidative reactions are prevented by cutting off the contact of pastırma with air (Turan & Şimşek, 2022). Fenugreek seed flour is influential on emulsions and rheological characteristics, owing to the galactomannan compounds. Adhesive and viscous solutions are formed due to galactomannans with high water‐binding capacity (Hegazy, 2011). Moreover, sweet red pepper used in çemen paste plays an important role in reducing the water activity on the meat surface, retarding the oxidative changes, preventing yeast–mold formation, and giving the desired red color and aroma to the product (Doğruer et al., 1998). Sweet red pepper contains high levels of ascorbic acid, provitamin A carotenoids, and antioxidant compounds, such as luteolin, phenolic acids, and tocopherols (Sánchez‐Escalante et al., 2003). On the other hand, garlic is an effective antimicrobial due to the allicin substance present in its content. Garlic also contributes to the formation of the unique taste and odor of çemen paste (Doğruer et al., 1998).

The purpose of the current research was to produce hamburgers containing different çemen paste formulations and investigate the impacts of çemen pastes on the physicochemical, microbiological, and textural properties of hamburgers during refrigerated storage.

## 2 MATERIALS AND METHODS

### 2.1 Materials

The 24 h post‐mortem fresh beef (*Longissimus thoracis et lumborum*, LL) and beef back fat were supplied from a local butcher (Isparta, Türkiye). Spices and fenugreek seed flour were purchased from Bağdat Baharat (Ankara, Türkiye).

### 2.2 Hamburger production

Hamburgers were produced, according to the procedure described by Kılıç et al. (2018). Hamburgers were produced in triplicate on separate production days. First, the meat was chopped by passing through 9.5 and 3.2 mm mincer chucks of a meat grinder (PKM‐22 Meat Grinder, Arı Machine, İstanbul), respectively. The beef back fat was minced using a meat grinder with 3.2 mm mincer chuck.

Hamburger patties were prepared, according to the formulations (%) for the production of hamburgers and group codes presented in Table 1. Differences in the hamburger formulation were sourced from applied ingredient doses. Çemen paste was prepared with a ratio of 1:1 mixture of water and çemen additives (fenugreek seed flour [FSF], garlic powder [GP], and sweet red pepper [SRP]). Hamburgers were shaped using a hamburger mold with a diameter of 10 cm and a thickness of 1 cm (approximately 100 g each). Hamburgers were cooked in a conventional oven (Kumtel, Türkiye) set at 200°C until the core temperature of 72°C. Hamburgers were cooled at ambient temperature for 20 min. The styrofoam trays (Alhas packaging, Türkiye) containing cooked hamburgers were placed into polyamide/polyethylene (PA/PE; 80 μm) pochettes with an O_2_ permeability of 12 cm^3^/(m^2^ 24h 1atm) and a water vapor permeability of 0.038 g mm/(h kPa m^2^). The pochettes were sealed by applying a vacuum of −0.85 bar for 10 s using a vacuum machine (Ramon VP280, Spain). The packaged hamburgers were stored at 4°C for 60 days. Proximate composition (moisture, fat, protein, and ash) and cooking characteristics (cooking loss, moisture and fat retention, dimensional shrinkage, decrease in patties diameter, and increase in patties thickness) were determined once for each of the three replications on production day, and the results of proximate composition and cooking characteristics are expressed as grams per one hundred grams (g/100 g) of hamburger in wet basis (%). pH, thiobarbituric acid reactive substances (TBARS), lipid hydroperoxide (LPO), and color values were determined on days 0, 15, 30, and 60 during storage. Texture profile, water activity (a_w_), and microbiological analyses were carried out on days 0, 30, and 60 during storage. Moreover, microbiological analyses were performed on raw hamburgers once.

**TABLE 1:** The formulations (%) for the production of hamburgers and group codes.

Ingredients (%)	Control	BC	CP1	CP2	CP3	CP4	CP5	CP6	CP7	CP8	CP9
Ground meat	76	68	67	67	67	67	67	67	67	67	67
Beef back fat	10	10	10	10	10	10	10	10	10	10	10
Potable water	10	10	10	10	10	10	10	10	10	10	10
Salt	2	2	2	2	2	2	2	2	2	2	2
Breadcrumbs	—	8	—	—	—	—	—	—	—	—	—
Onion powder	0.75	0.75	0.75	0.75	0.75	0.75	0.75	0.75	0.75	0.75	0.75
Black pepper	0.25	0.25	0.25	0.25	0.25	0.25	0.25	0.25	0.25	0.25	0.25
Fenugreek seed flour	—	—	5	4	3	4.5	3.5	2.5	4	3	2
Sweet red pepper	0.25	0.25	2.5	2.5	2.5	3	3	3	3.5	3.5	3.5
Garlic powder	0.75	0.75	2.5	3.5	4.5	2.5	3.5	4.5	2.5	3.5	4.5

### 2.3 Cooking characteristics and proximate composition

The percentage of cooking loss, cooking yield, moisture and fat retention were calculated according to the following equations expressed by López‐Vargas et al. (2014).

$$\begin{array}{c} \text{Cooking loss}\;\left(\%\right)\\ =\frac{\left(\;\text{weight of}\;\mathrm{raw}\;\text{hamburger}\right)-\left(\;\text{weight of cooked hamburger}\right)}{\left(\;\text{weight of}\;\mathrm{raw}\;\text{hamburger}\;\right)}\times 100. \end{array}$$

$$\text{Cooking yield}\;\left(\%\right)=\frac{\left(\;\text{cooked weight of hamburgers}\right)\;}{\left(\;\mathrm{raw}\;\text{weight of hamburgers}\right)}\times 100$$

Moisture retention (%) $=\frac{\text{percent cooking yield}\times \text{percent moisture in cooked hamburgers}\;}{100}$

$$\begin{array}{c} \mathrm{Fat}\;\text{retention}\;\left(\%\right)\\ =\frac{\left(\;\text{cooked weight of hamburgers}\right)\times \left(\;\text{percent}\;\mathrm{fat}\;\text{in cooked hamburgers}\right)}{\left(\;\mathrm{raw}\;\text{weight of hamburgers}\right)\times \left(\text{percent}\;\mathrm{fat}\;\text{in}\;\mathrm{raw}\;\text{hamburgers}\right)}\times 100 \end{array}$$

The diameters and thicknesses of raw and cooked hamburgers were measured from four different points using a digital caliper. The percentage of shrinkage, increase in thickness, and decrease in diameter were calculated according to the following equations described by Das et al. (2007).

$$\begin{array}{c} \text{Decrease in hamburger diameter}\;\left(\%\right)\\ =\frac{\left(\;\mathrm{raw}\;\text{hamburger diameter}-\text{cooked hamburger diameter}\right)}{\left(\;\mathrm{raw}\;\text{hamburger diameter}\right)}\times 100 \end{array}$$

$$\begin{array}{c} \text{Increase in hamburger thickness}\;\left(\%\right)\\ =\frac{\left(\;\text{Cooked hamburger diameter}-\mathrm{raw}\;\text{hamburger diameter}\right)}{\left(\;\text{cooked hamburger diameter}\right)}\times 100 \end{array}$$

$$\begin{array}{c} \text{Dimensional shrinkage}\;\left(\%\right)\\ =\frac{\left(\;\mathrm{Raw}\;\text{thickness}-\text{cooked thickness}\right)+\left(\;\mathrm{raw}\;\text{diameter}-\text{cooked diameter}\right)}{\left(\;\mathrm{raw}\;\text{thickness}+\mathrm{raw}\;\text{diameter}\right)}\times 100 \end{array}$$

Proximate composition, including moisture (950.46), fat (991.36), protein (992.15), and ash (920.153), measurements in raw and cooked hamburgers were performed, according to the Association of Official Analytical Chemists (AOAC (1997)) procedures.

### 2.4 Determination of pH, color, a_w_, TBARS, and LPO values

The pH was measured using a digital pH/ORP meter (Hanna HI 2211, Woonsocket, RI, USA) on homogenates resulting from blending a 10 g sample with 100 mL distilled water for 2 min. Color values (CIE L*, a*, and b*) were measured by choosing six random locations from inner surfaces. Color measurements were carried out by using a CR‐200 Chromameter (Illuminant D65, 8 mm aperture size, 10° standard observer, Minolta Corp., USA). The colorimeter was calibrated prior to use with black and white calibration plates (L* = 97.79, a* = −0.11, b* = 2.69). a_w_ was measured using a water activity device (Novasina LabSwift‐a_w_, Switzerland) at 25°C (López‐Vargas et al., 2014). TBARS and LPO measurements were carried out using the extraction procedures described by Kılıç et al. (2014). A standard curve was prepared using tetraethoxypropane for TBARS and cumene hydroperoxide for LPO measurements. TBARS and LPO values were expressed as *μ*mol malondialdehyde (MDA)/kg patty and *μ*mol LPO/kg patty, respectively.

### 2.5 Texture profile analysis (TPA)

Texture profile analysis (TPA) tests in hamburgers were performed using a CT3 Texture Analyzer (Brookfield Engineering Labs., Inc., USA). Hardness (N), adhesiveness (mJ), gumminess (N), cohesiveness, springiness, chewiness, and resilience values were determined with TPA (Caine et al., 2003). Hamburger samples were taken from the refrigerator and held at ambient temperature for 1 h. Hamburger samples were cut as 1 × 4 × 4 cm in size, placed on the texture analyzer's base, and subjected to texture profile analysis at three points for each of the three replications. Test conditions were: aluminum rectangular probe (9 mm × 35 mm × 0.05 mm), test speed 1 mm/s, pretest speed 1 mm/s, posttest speed 3 mm/s, compression 70%, and 25 kg load cell.

### 2.6 Microbiological analysis

The microbiological analyses were performed using methods described by Turan and Şimşek (2022). Plate Count Agar (PCA, Merck, Germany) for total mesophilic aerobic bacteria (TMAB), Eosin Methylene‐Blue Lactose Sucrose Agar (EMB, Merck, Germany) for total coliforms, and Potato Dextrose Agar (PDA, Merck, Germany) for yeasts–molds were used as selective agar medium. Inoculated PCA, PDA, and EMB agar mediums were incubated at 30°C for 24–48 h, 25°C for 72–120 h, and 37°C for 24–48 h, respectively. Microbiological analysis results were given as log colony‐forming units per gram ((CFU)/g) of hamburgers.

### 2.7 Design and statistical analysis

Statistical analysis was carried out by using the SPSS 18.0.0 software program (SPSS Inc., USA). All analyses were performed in triplicate for each of the three replications. The data obtained from analyses were subjected to one‐way analysis of variance (ANOVA). Significant differences between the means were evaluated by using Duncan's multiple range test. Moreover, the means obtained from proximate composition analyses of raw and cooked hamburgers were also separated, according to the two‐sample t‐test method. Differences among means were tested at 5% significance level. The results were given as mean ± standard deviation (SD).

## 3 RESULTS AND DISCUSSIONS

### 3.1 Cooking characteristics and proximate composition

Results (Table 2) demonstrated that the highest cooking loss (CL) value was determined in control (*p* < .05). Moreover, CL of hamburgers containing çemen paste did not significantly differ from breadcrumbs (BC) except CP1 which had lower CL than BC. Similarly, previous studies indicated that CL of meat products, such as beef hamburgers, beef meatballs, and spent hen meat patties, decreased due to BC or FSF usage (Ergezer et al., 2014; Hegazy, 2011; Qureshi et al., 2018). BC or FSF provides an excellent barrier against mass transfer from food matrix due to their adhesive properties (Hegazy, 2011; Putri et al., 2022). CP1 had a lower CL compared to control, BC, CP3, CP4, CP5, CP6, and CP7 (*p* < .05).

**TABLE 2:** Effects of breadcrumbs or çemen paste addition on the cooking characteristics (%) of hamburgers.

Groups	Cooking loss (%)	Moisture retention (%)	Fat retention (%)	Decrease in patty diameter (%)	Increase in patty thickness (%)	Shrinkage (%)
Control	28.50 ± 2.23^a^	45.49 ± 2.13^b^	40.31 ± 2.28^d^	23.65 ± 1.66^a^	33.63 ± 1.82^bc^	26.56 ± 1.46^a^
BC	16.82 ± 2.01^b^	50.99 ± 2.03^a^	47.48 ± 5.50^c^	20.59 ± 1.39^bcd^	22.40 ± 1.54^gh^	21.63 ± 1.81^de^
CP1	13.79 ± 2.34^c^	53.15 ± 3.61^a^	55.11 ± 3.44^b^	18.35 ± 1.84^d^	29.55 ± 2.13^cd^	21.35 ± 1.34^e^
CP2	16.09 ± 1.77^bc^	51.53 ± 1.93^a^	55.01 ± 5.10^b^	21.15 ± 1.25^abc^	36.92 ± 2.22^a^	25.74 ± 0.61^ab^
CP3	16.36 ± 3.53^b^	52.16 ± 3.17^a^	49.81 ± 7.61^bc^	19.45 ± 2.41^cd^	28.77 ± 3.44^de^	22.27 ± 1.95^de^
CP4	16.90 ± 3.36^b^	51.27 ± 3.11^a^	52.55 ± 4.41^bc^	20.14 ± 2.12^bcd^	28.65 ± 1.06^de^	22.72 ± 2.39^cde^
CP5	17.06 ± 2.95^b^	49.68 ± 3.08^a^	62.59 ± 5.66^a^	20.06 ± 1.57^bcd^	34.75 ± 0.94^ab^	24.01 ± 0.52^bcd^
CP6	17.09 ± 3.17^b^	51.34 ± 4.17^a^	50.68 ± 3.68^bc^	22.21 ± 1.25^ab^	29.78 ± 2.59^cd^	24.71 ± 1.78^abc^
CP7	17.24 ± 1.65^b^	51.52 ± 2.11^a^	55.04 ± 3.42^b^	20.44 ± 1.55^bcd^	24.75 ± 1.97^fg^	22.06 ± 2.32^de^
CP8	15.80 ± 2.04^bc^	52.49 ± 1.94^a^	49.55 ± 5.80^bc^	21.22 ± 2.47^abc^	20.49 ± 2.44^h^	21.82 ± 1.63^de^
CP9	15.80 ± 1.53^bc^	51.80 ± 2.33^a^	48.67 ± 2.66^bc^	20.34 ± 2.16^bcd^	26.33 ± 2.68^ef^	22.22 ± 1.51^de^

*Note*: Results are expressed as means ± SD; ^a–h^Means with different superscripts within a column are significantly different (*p* < .05).

Control had a lower moisture retention ratio compared to other groups, whereas there were no differences in terms of moisture retention ratios between BC and groups containing çemen paste. Likewise, Ergezer et al. (2014) noted that control had lower moisture retention ratio compared to the group containing BC (10%). Considering fat retention ratios, the highest fat retention ratio was determined in CP5 (*p* < .05). Conversely, the lowest fat retention ratio was obtained in control (*p* < .05). Moreover, CP1, CP2, and CP7 had higher fat retention ratios compared to BC (*p* < .05). Hegazy (2011) observed that hamburgers extended with 9 and 12% FSF had higher moisture and fat retention ratios. Additionally, Ergezer et al. (2014) revealed that the highest fat retention percentages were observed in the samples extended with 10% BC or 20% potato puree.

Control showed a greater decrease in hamburger diameter (*p* < 05) as a result of cooking compared to BC and groups with çemen paste except CP2, CP6, and CP8 which were quite similar to control. The reduction in diameter is related to the loss of water and fat depending on the denaturation of meat proteins (López‐Vargas et al., 2014). BC or çemen paste addition could have contributed to the reduction of this phenomenon through their water‐ and fat‐binding capacities. CP2, CP6, and CP8 had a higher decrease in hamburger diameter compared to CP1 (*p* < .05). Additionally, decreases in hamburger diameter were similar between BC and groups with çemen paste. Dinçer et al. (2018) reported that decrease in diameter of meatballs containing retrograded flour was lower, compared to those containing BC.

Increase in patty thickness values were lower (*p* < .05) in BC, CP3, CP4, CP7, CP8 and CP9 compared to control. This could be due to the binding properties of BC or çemen paste, which held both water and fat together and resisted dimensional changes of product. Das et al. (2007) demonstrated that soy paste addition reduced the ratio of increase in goat meat patty thickness. Furthermore, CP2 had a higher (*p* < .05) increased ratio in hamburger thickness compared to others (except CP5).

The shrinkage ratio of control was higher (*p* < .05) than those of other groups (except CP2 and CP6). Additionally, BC had a shrinkage ratio similar to those of groups with çemen paste (except CP2 and CP6). Similarly, Hegazy (2011) reported lower shrinkage ratios in hamburgers containing FSF at 9% and 12% levels with reference to that of control. Alqahtan et al. (2022) noted that the replacement of BC with bisr date powder at 50%, 75%, and 100% increased the dimensional shrinkage. Furthermore, Das et al. (2007) stated that soy paste addition in patties did not affect the dimensional shrinkage.

Proximate compositions of raw and cooked hamburgers are given in Table 3. Control had higher (*p* < .05) moisture contents than those of CP4 and CP8, whereas there were no differences between control and other groups. Other researchers reported that similar moisture changes were obtained as a result of using BC or FSF in beef patty (Ergezer et al., 2014; Hegazy, 2011). The cooking process did not influence moisture content in all groups (except CP8). Pinero et al. (2008) found a similar result for moisture content changes in beef patties due to the cooking process. In cooked hamburgers, CP5 had a lower moisture content compared to control, CP7, and CP8 (*p* < .05). In raw hamburgers, CP5 and CP7 had lower fat contents compared to control (*p* < .05). Likewise, Alqahtan et al. (2022) reported lower fat content in patties containing bisr date powder. Moreover, control had a lower (*p* < .05) protein content compared to other groups (except CP1, CP2, and CP8). Additionally, the lowest ash content in raw hamburgers was obtained in BC (*p* < .05). The ash content of CP2 was higher (*p* < .05) than those of raw hamburgers with çemen paste (except CP1) and control. Results demonstrated that fat contents in all groups decreased and their protein (except CP4, CP7, and CP8) and ash contents increased with cooking (*p* < .05). Similarly, Pinero et al. (2008) noted that there was a remarkable decrease in fat content of beef patties after the cooking process, and also, the protein and ash contents increased. In cooked hamburgers, BC had a lower fat content compared to control and CP5 (*p* < .05). Control had a lower (*p* < .05) protein content compared to groups of BC, CP1, CP3, CP5, CP6, and CP9. Qureshi et al. (2018) observed that the protein content of patties increased with FSF usage. Moreover, there were no ash content differences among groups of control, BC, CP3, and CP4, whereas BC had a lower ash content compared to those of CP1, CP2, CP5, CP6, CP7, CP8, and CP9 (*p* < .05). Ergezer et al. (2014) reported that there was no ash content difference between control and the patties containing BC.

**TABLE 3:** Effects of breadcrumbs or çemen paste addition on proximate composition (%) of hamburgers.

Groups	Moisture (%)		Fat (%)		Protein (%)		Ash (%)	
	Raw	Cooked	Raw	Cooked	Raw	Cooked	Raw	Cooked
Control	62.40 ± 2.71^aA^	63.60 ± 2.38^aA^	17.29 ± 1.75^aA^	9.64 ± 0.38^aB^	18.69 ± 0.97^eB^	23.17 ± 1.16^cA^	3.30 ± 0.10^bB^	3.76 ± 0.09^abA^
BC	60.55 ± 2.50^abA^	61.29 ± 1.40^bcA^	14.03 ± 3.20^abA^	7.66 ± 0.27^bB^	23.08 ± 3.26^a‐dB^	27.77 ± 0.31^aA^	3.11 ± 0.08^cB^	3.58 ± 0.08^bA^
CP1	60.76 ± 1.35^abA^	61.61 ± 2.73^abcA^	14.89 ± 1.67^abA^	9.42 ± 1.08^abB^	21.12 ± 2.03^b‐eB^	26.46 ± 1.82^abA^	3.41 ± 0.16^abB^	3.86 ± 0.13^aA^
CP2	61.56 ± 1.98^abA^	61.44 ± 2.79^abcA^	14.58 ± 1.27^abA^	9.46 ± 1.08^abB^	20.71 ± 1.84^deB^	25.69 ± 1.76^abcA^	3.56 ± 0.12^aB^	3.94 ± 0.16^aA^
CP3	60.94 ± 2.50^abA^	62.33 ± 1.28^abA^	14.23 ± 1.61^abA^	8.25 ± 1.02^abB^	23.29 ± 0.91^abcB^	26.13 ± 1.73^abA^	3.32 ± 0.16^bB^	3.74 ± 0.12^abA^
CP4	59.97 ± 3.32^bA^	61.68 ± 2.23^abcA^	14.20 ± 0.85^abA^	8.83 ± 1.82^abB^	24.89 ± 0.98^aA^	25.66 ± 3.20^abcA^	3.35 ± 0.12^bB^	3.78 ± 0.13^abA^
CP5	61.50 ± 2.05^abA^	59.88 ± 2.68^cA^	13.33 ± 0.89^bA^	9.93 ± 1.83^aB^	22.89 ± 1.03^a‐dB^	27.12 ± 0.99^abA^	3.37 ± 0.08^bB^	3.80 ± 0.18^aA^
CP6	60.97 ± 2.41^abA^	61.85 ± 2.70^abcA^	14.84 ± 2.97^abA^	8.78 ± 0.08^abB^	22.10 ± 1.20^bcdB^	27.12 ± 1.81^abA^	3.37 ± 0.12^bB^	3.80 ± 0.23^aA^
CP7	61.53 ± 2.89^abA^	62.27 ± 2.64^abA^	13.51 ± 2.00^bA^	8.66 ± 1.75^abB^	23.65 ± 1.59^abA^	25.14 ± 2.40^bcA^	3.36 ± 0.13^bB^	3.89 ± 0.13^aA^
CP8	59.66 ± 1.97^bB^	62.34 ± 1.92^abA^	16.58 ± 4.48^abA^	8.98 ± 1.83^abB^	21.01 ± 2.65^cdeA^	24.91 ± 1.81^bcA^	3.32 ± 0.10^bB^	3.79 ± 0.10^aA^
CP9	59.74 ± 2.28^bA^	61.50 ± 2.20^abcA^	15.49 ± 2.84^abA^	8.71 ± 0.48^abB^	22.78 ± 1.52^a‐dB^	27.20 ± 1.41^abA^	3.29 ± 0.13^bB^	3.79 ± 0.14^aA^

*Note*: Results are expressed as means±SD; ^a–e^Means with different superscripts within a column are significantly different (*p* < .05). ^A,B^Considering the different cooking states for each of the proximate compositions; means with different superscripts within a row are significantly different (*p* < .05).

### 3.2 pH, color, water activity, and texture profile analysis

As shown in Table 4, the highest pH was obtained in control and BC groups on day 0, whereas the lowest pH was determined in CP7, CP8, and CP9 (*p* < .05). Patriani et al. (2023) noted that FSF addition to buffalo meat patty caused a decrease in pH. The authors also stated that the rate of pH decrease caused by FSF addition was more evident with the increasing FSF dose incorporated. Similarly, Hegazy (2011) stated that FSF usage in hamburgers eventuated a lower pH. Conversely, Qureshi et al. (2018) indicated that the use of FSF in spent hen meat patties caused higher pH values. Furthermore, Martínez, Cilla, et al. (2006a) reported that SRP usage in pork sausages did not cause pH differences. Additionally, previous studies showed that the addition of garlic powder or black garlic powder into fresh pork loin or spent duck meat nuggets resulted in lower pH values (Park et al., 2008; Lishianawati et al., 2022). Even though garlic powder is generally considered as an alkaline ingredient, the acidity of garlic powder has been speculated to show variation. For instance, Hwang and Kim (2022) reported that pH of the commercial garlic powders ranged from 5.78 to 6.85. On the other hand, Sun et al. (2000) noted that fresh garlic addition to Chinese sausages had an increasing effect on pH, whereas the added garlic powder did not cause a significant change. Similarly, Kim et al. (2019) stated that fresh garlic incorporation into pork patties increased pH values. Moreover, BC had a lower (*p* < .05) pH compared to CP1, CP3, CP5, and CP8 groups on day 15. pH values generally increased in all groups (except control and BC) during the first 15 days of storage (*p* < .05). An increase in pH may be due to protein degradation that occurred as a result of the dissociation of bonds containing hydroxyl, sulfhydryl, and imidazole groups (Khan et al., 2019). Conversely, significant pH decreases (*p* < .05) were observed on days 30 and 60 for all groups. Similarly, Yingyuad et al. (2006) observed that pH values of vacuum‐packed grilled pork gradually decreased during storage. The major factor in pH decrease in vacuum‐packed meat products is the growth of lactic acid bacteria resulting in lactic acid production (Fernández‐López et al., 2008). In the present study, control had a lower pH than those of CP1, CP2, CP3, and CP5, and a higher pH than those of BC, CP6, CP7, and CP9 on day 30 (*p* < .05). On day 60, the lowest pH value was obtained in BC, whereas control had the highest pH value (*p* < .05).

**TABLE 4:** Effects of breadcrumbs or çemen paste addition on pH values of hamburgers.

Groups	pH			
	0 d	15 d	30 d	60 d
Control	6.10 ± 0.03^aA^	6.14 ± 0.10^abA^	5.64 ± 0.02^cB^	5.51 ± 0.08^aC^
BC	6.12 ± 0.02^aA^	6.10 ± 0.01^bA^	5.51 ± 0.10^defB^	4.94 ± 0.07^eC^
CP1	6.04 ± 0.03^bB^	6.15 ± 0.02^aA^	5.82 ± 0.14^aC^	5.34 ± 0.03^bcdD^
CP2	6.04 ± 0.04^bB^	6.14 ± 0.03^abA^	5.80 ± 0.17^abC^	5.36 ± 0.07^bcD^
CP3	6.03 ± 0.02^bB^	6.18 ± 0.03^aA^	5.74 ± 0.08^abC^	5.32 ± 0.08^bcdD^
CP4	6.04 ± 0.02^bB^	6.14 ± 0.03^abA^	5.59 ± 0.09^cdC^	5.35 ± 0.03^bcD^
CP5	6.02 ± 0.03^bB^	6.16 ± 0.02^aA^	5.73 ± 0.05^bC^	5.41 ± 0.11^bD^
CP6	6.02 ± 0.02^bB^	6.14 ± 0.04^abA^	5.47 ± 0.05^fC^	5.31 ± 0.21^cdD^
CP7	5.98 ± 0.03^cB^	6.14 ± 0.04^abA^	5.53 ± 0.03^defC^	5.41 ± 0.10^bD^
CP8	5.97 ± 0.03^cB^	6.16 ± 0.05^aA^	5.58 ± 0.06^cdeC^	5.27 ± 0.11^cdD^
CP9	5.97 ± 0.03^cB^	6.14 ± 0.03^abA^	5.50 ± 0.03^efC^	5.25 ± 0.04^dD^

*Note*: Results are expressed as means ± SD; ^a–f^Means with different superscripts within a column are significantly different (*p* < .05). ^A–D^Means with different superscripts within a row are significantly different (*p* < .05).

Color results (Table 5) showed that the highest L* values on days 0 and 15 were obtained in control compared to other groups (*p* < .05). Moreover, control and BC had the highest (*p* < .05) L* values during the rest of the storage. Hamburgers containing çemen paste had lower L* values compared to control and BC in all storage days (*p* < .05). Several researchers have noted L* values decrease due to retrograded and cocoa shell flour usage in patties (Delgado‐Ospina et al., 2022; Dinçer et al., 2018). There was no difference in L* among groups containing çemen paste on day 0. L* value of CP1 on day 15 was higher than those of CP4, CP7, and CP8, whereas L* values of CP3 on days 30 and 60 were higher than those of CP4 and CP7 (*p* < .05). Moreover, L* values of all groups (except control, CP4, and CP6) on day 60 generally increased (*p* < .05) compared to production day. These increases in L* values might have been caused by myoglobin oxidation during storage (Fernández‐López et al., 2008).

**TABLE 5:** Effects of breadcrumbs or çemen paste addition on CIE L* a* b* values of hamburgers.

Groups	CIE L*			
	0 d	15 d	30 d	60 d
Control	52.45 ± 2.93^aA^	51.18 ± 0.84^aAB^	52.38 ± 2.93^aA^	51.31 ± 1.33^aA^
BC	49.51 ± 1.61^bB^	49.17 ± 1.15^bB^	51.18 ± 1.73^aA^	51.67 ± 1.82^aA^
CP1	44.41 ± 1.87^cB^	45.56 ± 1.43^cAB^	45.55 ± 1.42^bcAB^	46.63 ± 1.54^bcA^
CP2	44.24 ± 3.29^cB^	44.85 ± 1.23^cdeAB^	46.66 ± 1.71^bA^	46.20 ± 1.42^bcA^
CP3	43.32 ± 3.04^cB^	44.47 ± 1.47^c‐fB^	46.82 ± 1.39^bA^	47.15 ± 1.26^bA^
CP4	44.61 ± 2.95^cA^	43.65 ± 1.62^efA^	44.36 ± 1.60^cdA^	45.40 ± 1.12^cA^
CP5	43.66 ± 0.90^cC^	44.73 ± 0.86^cdeBC^	45.72 ± 0.99^bcAB^	46.60 ± 1.55^bcA^
CP6	44.69 ± 2.19^cA^	44.69 ± 1.21^cdeA^	46.20 ± 1.10^bA^	46.23 ± 1.60^bcA^
CP7	43.52 ± 2.04^cB^	43.32 ± 2.07^fB^	43.51 ± 1.65^dB^	45.45 ± 1.59^cA^
CP8	44.67 ± 0.90^cBC^	44.14 ± 0.62^defC^	45.56 ± 1.33^bcAB^	46.50 ± 1.09^bcA^
CP9	43.90 ± 1.76^cB^	45.26 ± 1.66^cdAB^	45.71 ± 1.22^bcAB^	46.71 ± 1.21^bcA^

Groups	CIE a*			
	0 d	15 d	30 d	60 d
Control	13.64 ± 1.61^bcAB^	12.52 ± 1.00^efB^	13.87 ± 0.80^abA^	13.61 ± 0.61^bcA^
BC	10.64 ± 0.60^eB^	10.97 ± 0.91^gB^	11.66 ± 1.06^cAB^	12.49 ± 1.62^cA^
CP1	11.41 ± 0.89^deB^	11.34 ± 0.82^gB^	12.94 ± 1.27^bcA^	13.79 ± 1.34^abcA^
CP2	12.99 ± 2.20^bcAB^	11.80 ± 0.77^fgB^	13.28 ± 1.30^abcA^	13.50 ± 1.68^bcA^
CP3	12.32 ± 1.38^cdA^	12.44 ± 0.35^efA^	13.01 ± 1.19^bcA^	13.24 ± 2.59^bcA^
CP4	13.07 ± 1.36^bcB^	14.68 ± 1.37^bA^	14.87 ± 1.25^aA^	14.40 ± 2.26^abAB^
CP5	13.87 ± 1.77^bcA^	13.90 ± 0.86^bcA^	14.44 ± 2.30^abA^	14.73 ± 1.24^abA^
CP6	14.29 ± 1.19^abAB^	12.98 ± 0.99^deB^	14.85 ± 2.17^aA^	14.52 ± 1.67^abAB^
CP7	13.50 ± 1.42^bcA^	13.91 ± 0.42^bcA^	14.64 ± 1.66^aA^	14.13 ± 1.49^abcA^
CP8	14.13 ± 1.77^bA^	13.61 ± 0.93^cdA^	14.82 ± 2.54^aA^	14.69 ± 1.38^abA^
CP9	15.82 ± 2.16^aA^	16.08 ± 1.09^aA^	14.00 ± 1.48^abA^	15.50 ± 3.57^aA^

Groups	CIE b*			
	0 d	15 d	30 d	60 d
Control	5.03 ± 1.91^eA^	5.12 ± 0.76^fA^	4.15 ± 0.95^gA^	4.55 ± 1.16^fA^
BC	8.00 ± 0.69^dA^	6.76 ± 0.72^eB^	6.46 ± 1.18^fBC^	5.81 ± 1.02^fC^
CP1	13.75 ± 1.07^cA^	12.95 ± 1.54^cdAB^	12.35 ± 1.29^eB^	13.11 ± 1.28^dAB^
CP2	13.38 ± 1.38^cA^	12.63 ± 1.16^dA^	12.86 ± 1.06^deA^	10.93 ± 0.76^eB^
CP3	15.92 ± 3.05^abA^	13.19 ± 1.13^cdB^	13.15 ± 1.60^cdeB^	13.79 ± 1.28^cdB^
CP4	14.80 ± 1.64^bcA^	13.44 ± 2.54^bcdA^	14.44 ± 1.71^abcA^	12.88 ± 1.83^dA^
CP5	17.10 ± 1.75^aA^	14.91 ± 1.60^abB^	14.36 ± 1.64^abcB^	15.32 ± 1.23^bB^
CP6	15.97 ± 2.12^abA^	14.26 ± 2.20^bcAB^	13.88 ± 1.51^bcdB^	15.17 ± 1.90^bcAB^
CP7	15.88 ± 1.54^abA^	14.16 ± 1.45^bcdB^	13.49 ± 1.18^b‐eB^	14.99 ± 1.70^bcAB^
CP8	16.78 ± 1.03^aA^	16.07 ± 1.54^aAB^	14.87 ± 1.27^abB^	16.77 ± 1.17^aA^
CP9	17.03 ± 1.16^aA^	16.21 ± 2.08^aA^	15.50 ± 1.81^aA^	16.30 ± 1.73^abA^

*Note*: Results are expressed as means ± SD; ^a–g^Means with different superscripts within a column are significantly different (*p* < .05). ^A–C^Means with different superscripts within a row are significantly different (*p* < .05).

Regarding a* values (Table 5), CP9 had higher (*p* < .05) a* values on days 0 and 15 than other groups (except CP6 on day 0). This result is thought to be related to higher SRP and lower FSF content in CP9 formulation. Similar results were demonstrated by Martínez, Cilla, et al. (2006a) who noted that SRP addition to sausages increased a* values. This effect is due to the high carotenoid content of SRP, mainly ketocarotenoids such as capsanthin and capsorubin (Martínez, Cilla, et al., 2006a). On day 30, there were no significant a* value differences between control and groups containing çemen paste. Moreover, BC had generally lower (*p* < .05) a* value than those of other groups (except CP1 on day 0, CP1 and CP2 on day 15, and CP1, CP2, and CP3 on day 30) during the first 30 days of storage. Similarly, Ergezer et al. (2014) observed that control had higher a* values compared to meatballs containing BC, which was associated with the dilution of the meat pigment due to BC. a* value of CP9 on day 60 was higher than those obtained in control, BC, CP2, and CP3 (*p* < .05). Considering the storage period, the groups (except BC and CP1) did not generally exhibit a significant change in a* values during storage. a* value changes determined in this study were similar to those noted by several researchers (Cachaldora et al., 2013; Martínez, Djenane, et al., 2006b).

Color results (Table 5) indicated that the lowest b* values were observed in control during the first 30 days of storage followed by those of BC (*p* < .05). On day 60, control and BC had the lowest b* values (*p* < .05). These results showed that çemen paste usage in hamburgers increased b* values (*p* < .05). Similar results were reported for increase in b* values in sausages containing SRP (Martínez, Cilla, et al., 2006a) and meatballs containing fresh or aged garlic (Kim et al., 2019). Results also indicated that CP8 and CP9 groups had generally higher b* values than those of CP1, CP2, CP3, and CP4 (*p* < .05). b* values of control, CP1, CP4, CP6, CP7, CP8, and CP9 were generally stable during storage. Likewise, Kök and Çiçek (2022) observed that there was no significant change in b* values of meatballs containing fenugreek paste during the 5 days of processing stage. Additionally, b* values of BC gradually decreased during storage (*p* < .05). Moreover, b* values of CP2 decreased during the duration of day 30 to day 60, whereas b* values decreased on day 15 compared to day 0 for groups of CP3 and CP5 (*p* < .05).

There was no a_w_ difference among groups (data not shown) on day 0. Dinçer et al. (2018) reported that there was no a_w_ difference among meatballs containing bread, rusk, or retrograded flour. On day 30, CP2, CP5, and CP6 had lower a_w_ compared to CP7 (*p* < .05). Moreover, control had the highest a_w_ on day 60 (*p* < .05). a_w_ values did not differ between BC and groups containing çemen paste (except CP7 on day 60) in all storage days. a_w_ values of groups (except control and CP5) decreased on day 60 compared to production day (*p* < .05).

The TPA results (data not shown) revealed that the highest hardness (N), resilience, cohesiveness, gumminess (N), and chewiness values were determined in control (*p* < .05). These results showed that the addition of çemen paste into hamburger patty formulation resulted in a decrease in textural parameters (*p* < .05). Likewise, previous studies indicated that the addition of various ingredients, such as soy paste, flaxseed flour, or tomato paste into goat or beef patties, decreased the hardness, springiness, cohesiveness, chewiness, and gumminess values (Das et al., 2007; Valenzuela‐Melendres et al., 2018). Additionally, Bastos et al. (2014) found that hamburgers containing various types of flour presented lower hardness. As these flours or pastes are fiber sources, they contribute to hygroscopicity and thus provide the characteristic juiciness of the final product. There were no significant differences between control and BC groups in terms of adhesiveness (N sec) and springiness. BC had generally higher (*p* < .05) hardness (N), gumminess (N), and chewiness values compared to groups containing çemen paste (except CP4, CP6, and CP7). Resilience values of groups containing çemen paste were lower than those of BC (*p* < .05). Conversely, there were no differences in cohesiveness values between BC and groups containing çemen paste. Alqahtan et al. (2022) reported that hamburgers containing BC had higher hardness, gumminess, and chewiness values compared to hamburgers containing bisr date powder. No significant differences were generally obtained among groups with çemen paste in terms of adhesiveness (N sec), resilience, cohesiveness, gumminess (N), and chewiness. CP2 had generally lower hardness (N) value compared to CP4, CP6, and CP7 (*p* < .05). TPA showed a gradual increase (*p* < .05) in hardness (N) values during storage in groups (except control and CP5), whereas resilience and cohesiveness values of all groups were quite stable during storage. Adhesiveness (N sec), springiness, gumminess (N), and chewiness values of groups generally increased on day 60 compared to production day (*p* < .05).

### 3.3 Thiobarbituric acid reactive (TBARS) substances and lipid hydroperoxide

According to thiobarbituric acid reactive (TBARS) results (Table 6), control and BC displayed similar TBARS levels on day 0 to groups containing çemen paste (except CP1, CP4, and CP7). On days 0 and 15, CP1, CP4, and CP7 displayed generally higher TBARS levels than those of control and BC (*p* < .05). TBARS levels were similar between control and other groups (except CP1, CP7, and CP9) on day 15. Moreover, TBARS levels were lower in CP9 and higher in CP1 and CP7 compared to control on day 15 (*p* < .05). Whereas TBARS levels of control were similar to those of CP1, CP2, CP4, CP7, and CP8 on day 30 and as regards CP1 and CP7 on day 60, TBARS levels of control were higher than those of others (*p* < .05). Hegazy (2011) revealed that hamburgers containing 3% FSF had higher TBA content compared to control, whereas an increase of FSF in hamburgers from 6% to 12% resulted in a high TBA reduction. Pandey and Awasthi (2015) reported that FSF has antioxidant activity due to its high content of flavonoids and phenolic compounds. On days 30 and 60, CP9 (2% FSF, 3.5% SRP, and 4.5% GP) had generally lower TBARS compared to those of other groups (except BC and CP6 on day 30, and CP6 on day 60). Consistent with these results, previous studies demonstrated that SRP and fresh or aged garlic were remarkably effective in inhibiting lipid oxidation in beef and pork patties (Kim et al., 2019; Sánchez‐Escalante et al., 2003). Study results revealed a gradual increase in TBARS in all groups during storage (*p* < .05). Likewise, previous studies revealed that TBA values of patties containing BC or çemen paste increased during the processing or storage period (Ergezer et al., 2014; Kök & Çiçek, 2022).

**TABLE 6:** Effects of breadcrumbs or çemen paste addition on TBARS and LPO levels of hamburgers.

Groups	TBARS (μmol MDA/kg)			
	0 d	15 d	30 d	60 d
Control	3.55 ± 0.31^bD^	5.39 ± 0.23^cdC^	7.42 ± 0.37^aB^	10.59 ± 0.17^aA^
BC	3.44 ± 0.20^bD^	5.03 ± 0.50^deC^	5.89 ± 0.39^cdB^	8.11 ± 0.17^deA^
CP1	4.01 ± 0.32^aD^	5.95 ± 0.54^abC^	7.08 ± 0.55^aB^	10.14 ± 0.61^abA^
CP2	3.86 ± 0.13^abD^	5.03 ± 0.52^deC^	6.81 ± 0.16^abB^	9.32 ± 0.31^bcA^
CP3	3.77 ± 0.20^abD^	5.24 ± 0.27^cdC^	6.22 ± 0.67^bcB^	7.48 ± 0.29^efA^
CP4	4.08 ± 0.32^aD^	5.71 ± 0.49^abcC^	6.98 ± 0.32^aB^	8.97 ± 0.21^cdA^
CP5	3.46 ± 0.28^bC^	5.46 ± 0.31^bcdB^	6.02 ± 0.61^cB^	8.16 ± 0.59^deA^
CP6	3.75 ± 0.20^abD^	4.97 ± 0.20^deC^	5.85 ± 0.15^cdB^	7.92 ± 0.74^efA^
CP7	4.10 ± 0.31^aD^	6.14 ± 0.35^aC^	7.29 ± 0.73^aB^	9.91 ± 0.61^abA^
CP8	3.84 ± 0.37^abD^	5.50 ± 0.23^bcdC^	7.12 ± 0.74^aB^	8.84 ± 0.91^cdA^
CP9	3.56 ± 0.45^bC^	4.62 ± 0.23^eB^	5.24 ± 0.55^dB^	7.11 ± 0.90^fA^

	Lipid hydroperoxide (μmol LPO/kg)			
Groups	0 d	15 d	30 d	60 d
Control	46.13 ± 3.62^aC^	71.61 ± 4.98^abB^	90.74 ± 8.02^aB^	129.75 ± 10.81^aA^
BC	44.55 ± 0.92^abC^	66.98 ± 2.57^abcBC^	84.19 ± 8.54^abcB^	118.6 ± 14.8^abA^
CP1	41.40 ± 1.70^abcC^	72.48 ± 8.64^aB^	87.19 ± 9.73^abB^	126.97 ± 9.78^aA^
CP2	38.59 ± 3.78^abcD^	57.25 ± 6.15^cdC^	76.67 ± 6.19^abcB^	107.81 ± 7.69^bcA^
CP3	40.55 ± 4.67^abcC^	67.73 ± 6.45^abcB^	78.69 ± 8.07^abcB^	104.40 ± 6.09^bcA^
CP4	37.44 ± 2.60^bcD^	53.81 ± 5.26^dC^	71.51 ± 6.89^cB^	98.38 ± 7.14^cA^
CP5	40.34 ± 2.97^abcD^	59.98 ± 2.56^bcdC^	76.42 ± 5.93^abcB^	102.33 ± 3.60^bcA^
CP6	38.67 ± 4.69^abcD^	56.20 ± 4.60^cdC^	75.52 ± 3.73^abcB^	93.21 ± 8.18^cA^
CP7	42.19 ± 5.45^abcC^	64.84 ± 6.28^a‐dB^	79.23 ± 7.76^abcB^	106.15 ± 4.36^bcA^
CP8	37.31 ± 4.44^bcC^	62.02 ± 4.78^a‐dB^	73.90 ± 4.22^bcB^	95.93 ± 8.18^cA^
CP9	36.39 ± 2.28^cD^	56.58 ± 5.50^cdC^	74.35 ± 5.72^bcB^	91.50 ± 6.59^cA^

*Note*: Results are expressed as means ± SD; ^a–f^Means with different superscripts within a column are significantly different (*p* < .05). ^A–D^Means with different superscripts within a row are significantly different (*p* < .05).

The LPO results (Table 6) showed that control had generally higher LPO levels than those of CP4, CP8, and CP9 during storage (*p* < .05). Conversely, there were no LPO differences between control and BC during storage. Moreover, LPO levels were generally similar between BC and groups containing çemen paste (except CP9 on day 0, and CP4 on day 15) during the first 30 days of storage. On day 60, the groups of CP4, CP6, CP8, and CP9 had lower LPO compared to BC (*p* < .05). LPO results indicated that there were generally no LPO differences among groups containing çemen paste (except CP1) on days 0, 30, and 60. Conversely, CP1 had higher LPO levels compared to CP2, CP4, CP5, CP6, and CP9 on day 15, CP4 on day 30, and all other groups containing çemen paste on day 60 (*p* < .05). Moreover, LPO levels of all groups gradually increased during storage (*p* < .05). Das et al. (2011) revealed a significantly linear increase in peroxide value (POV) of cooked ground goat meat during storage.

### 3.4 Microbiological analysis results

Microbiological analysis results of raw and cooked hamburgers are shown in Table 7. The groups containing çemen paste (except CP9) in raw hamburgers displayed TMAB counts similar to control and BC. Likewise, previous studies showed that there were no significant differences in total bacterial counts between control and the patties containing SRP or GP (Mancini et al., 2020; Shim & Chin, 2013). Regarding total coliforms in raw hamburgers, control had a higher (*p* < .05) coliform count compared to BC and CP6. Conversely, higher coliform counts were determined in CP1, CP3, and CP4 compared to control (*p* < .05). According to yeast–mold counts in raw hamburgers, control, BC, CP1, and CP2 had lower yeast–mold counts compared to other groups (*p* < .05). Previous studies showed that initial TMAB, coliform, and yeast–mold counts of raw hamburgers or meatballs ranged from 6.05 to 7.48 log CFU/g, 3.77 to 3.94 log CFU/g, and 3.09 to 4.33 log CFU/g, respectively (Barbosa et al., 2022; Mancini et al., 2020; Yılmaz et al., 2005). The authors emphasized that the microbial load of raw hamburgers or meatballs might be related to the meat grinding process, or the addition of spices and other ingredients into minced meat. In the present study, the counts of TMAB, total coliform, and yeast–mold in all groups (except CP1 and CP2 for total coliforms) decreased as a result of the cooking process (*p* < .05). Yılmaz et al. (2005) noted that initial TMAB load of meatballs decreased by approximately 2–3 log cycles after grilling or oven cooking processes. Lahou et al. (2015) reported that the heating rate applied, nutritional composition (e.g., fat content) of meat material used, and the protective impact of some ingredients due to pH alteration or adaptation capability of microorganisms to stress conditions might have significantly influenced the survival of microorganisms in heat‐treated meat products.

**TABLE 7:** Effects of breadcrumbs or çemen paste addition on microbiological counts of hamburgers.

Groups	Total mesophilic aerobic bacteria counts (log_10_ CFU/g)			
	Raw hamburger	0 d	30 d	60 d
Control	6.06 ± 0.86^bcA^	4.54 ± 0.89^abB^	6.96 ± 0.11^aA^	7.13 ± 0.51^bcA^
BC	5.72 ± 0.43^cB^	4.35 ± 0.76^abC^	7.03 ± 0.02^aA^	6.27 ± 0.91^cAB^
CP1	5.89 ± 0.61^bcA^	4.82 ± 0.84^aB^	6.56 ± 0.05^abcA^	6.49 ± 1.38^bcA^
CP2	5.72 ± 0.36^cA^	4.40 ± 1.06^abB^	6.19 ± 0.07^cA^	6.72 ± 0.98^bcA^
CP3	6.54 ± 0.38^abA^	3.70 ± 0.03^bB^	6.82 ± 0.01^abA^	6.70 ± 0.28^bcA^
CP4	5.99 ± 0.41^bcB^	3.90 ± 0.88^bC^	7.07 ± 0.08^aA^	6.46 ± 0.92^bcAB^
CP5	6.01 ± 0.34^bcB^	4.36 ± 0.26^abC^	6.63 ± 0.04^abcA^	6.60 ± 0.62^bcA^
CP6	5.87 ± 0.33^cB^	4.29 ± 0.42^abC^	7.00 ± 0.54^aA^	6.42 ± 0.52^cAB^
CP7	6.08 ± 0.41^bcB^	4.87 ± 0.33^aC^	6.30 ± 0.14^bcB^	7.55 ± 0.96^bA^
CP8	5.60 ± 0.26^cC^	4.91 ± 0.08^aD^	6.70 ± 0.14^abcB^	8.83 ± 0.06^aA^
CP9	7.10 ± 0.36^aA^	5.04 ± 0.34^aB^	6.70 ± 1.05^abcA^	6.57 ± 0.34^bcA^

Groups	Total coliforms (log_10_ CFU/g)			
	Raw hamburger	0 d	30 d	60 d
Control	4.84 ± 0.22^cdA^	2.99 ± 0.53^cBC^	3.46 ± 0.22^abB^	2.73 ± 0.61^eC^
BC	4.58 ± 0.08^efA^	3.38 ± 0.44^bcB^	3.26 ± 0.61^bB^	3.61 ± 0.58^cdB^
CP1	5.05 ± 0.32^abA^	4.36 ± 0.42^aAB^	3.91 ± 0.89^abB^	4.38 ± 0.10^abAB^
CP2	4.66 ± 0.24^defA^	3.50 ± 0.34^abcA^	4.11 ± 1.06^abA^	3.44 ± 0.97^dA^
CP3	5.24 ± 0.06^aA^	3.38 ± 0.92^bcB^	3.34 ± 1.30^abB^	3.79 ± 0.44^bcdB^
CP4	5.05 ± 0.03^abA^	2.75 ± 0.31^cB^	3.42 ± 1.32^abB^	4.55 ± 0.12^aA^
CP5	4.79 ± 0.03^cdeA^	3.00 ± 0.10^cB^	3.24 ± 1.01^bB^	3.76 ± 0.23^cdB^
CP6	4.55 ± 0.03^fA^	2.94 ± 0.46^cB^	4.38 ± 1.21^abA^	4.21 ± 0.19^abcA^
CP7	4.92 ± 0.11^bcA^	4.00 ± 0.89^abB^	4.57 ± 0.61^aAB^	4.51 ± 0.15^aAB^
CP8	4.70 ± 0.06^defA^	3.36 ± 1.31^bcB^	4.09 ± 0.46^abAB^	4.17 ± 0.07^abcAB^
CP9	4.74 ± 0.06^c‐fA^	4.12 ± 0.44^abB^	4.51 ± 0.38^abAB^	4.57 ± 0.17^aA^

Groups	Yeast–mold counts (log_10_ CFU/g)			
	Raw hamburger	0 d	30 d	60 d
Control	3.87 ± 0.47^bA^	2.75 ± 0.87^aB^	1.42 ± 0.49^cC^	1.27 ± 0.32^bC^
BC	4.01 ± 0.46^bA^	1.76 ± 0.54^bcdB^	1.46 ± 0.55^cB^	<1^cC^
CP1	3.81 ± 0.52^bA^	2.24 ± 0.28^abcB^	<1^dC^	1.78 ± 0.90^bB^
CP2	4.02 ± 0.45^bA^	1.41 ± 0.14^dC^	2.51 ± 0.59^aB^	1.56 ± 0.65^bC^
CP3	4.98 ± 0.27^aA^	1.25 ± 0.33^dB^	1.70 ± 0.82^bcB^	1.70 ± 0.81^bB^
CP4	5.35 ± 0.21^aA^	1.44 ± 0.51^dC^	2.30 ± 0.35^abB^	2.08 ± 0.10^bB^
CP5	5.30 ± 0.23^aA^	1.27 ± 0.32^dB^	1.88 ± 1.02^abcB^	1.71 ± 0.82^bB^
CP6	5.09 ± 0.45^aA^	1.57 ± 0.44^cdC^	1.49 ± 0.56^cC^	2.98 ± 0.70^aB^
CP7	5.49 ± 0.32^aA^	2.45 ± 0.52^abB^	<1^dC^	2.03 ± 0.31^bB^
CP8	5.31 ± 0.23^aA^	2.26 ± 0.30^abcC^	<1^dD^	2.91 ± 0.48^aB^
CP9	5.27 ± 0.15^aA^	2.63 ± 0.73^aB^	<1^dD^	1.69 ± 0.34^bC^

*Note*: Results are expressed as means ± SD; ^a–f^Means with different superscripts within a column are significantly different (*p* < .05). ^A–D^Means with different superscripts within a row are significantly different (*p* < .05).

In cooked hamburgers, control and BC had TMAB counts similar to those of groups containing çemen paste (except CP2 and CP7 on day 30, and CP7 and CP8 on day 60) throughout the storage. TMAB counts of CP2 and CP7 were lower than those of control, BC, CP4, and CP6 on day 30 (*p* < .05). On day 60, the highest TMAB count was obtained in CP8 (*p* < .05). Moreover, TMAB counts of cooked hamburgers increased during the first 30 days of storage (*p* < .05). Contrarily, TMAB counts of groups (except CP7 and CP8) did not change during the rest of the storage. According to total coliforms on day 0, CP1 had a higher coliform count than control and BC (*p* < .05). There were no coliform count differences between BC and groups containing çemen paste (except CP1) on day 0. Generally, no significant coliform count difference was determined among groups on day 30. The lowest coliform count was obtained in control on day 60 (*p* < .05). Moreover, BC had lower coliform count compared to CP1, CP4, CP7, and CP9 (*p* < .05). Coliform counts only determined in CP4, CP6, and CP9 increased on day 60 compared to production day (*p* < .05). On day 0, yeast–mold count of control was higher than those of BC, CP2, CP3, CP4, CP5, and CP6 (*p* < .05). CP2 and CP4 groups had higher (*p* < .05) yeast–mold counts on day 30 compared to BC and control. Moreover, the lowest yeast–mold counts on day 30 were determined in CP1, CP7, CP8, and CP9 (*p* < .05). There was generally no significant yeast–mold count difference among groups (except BC) on day 60. Yeast–mold counts obtained from CP4, CP6, and CP8 increased on day 60 compared to production day, whereas yeast–mold counts of control, BC, and CP9 decreased on day 60 (*p* < .05).

## 4 CONCLUSIONS

Study results showed that çemen paste addition generally reduced cooking loss and dimensional shrinkage, and increased moisture and fat retention ratios of hamburgers. Çemen paste usage generally did not cause a difference in the moisture, fat, and ash contents of hamburgers. Conversely, the protein contents of BC and groups containing high amounts of FSF or garlic in hamburgers were found to be higher than in control. Çemen paste addition to hamburgers generally reduced the hardness, gumminess, and chewiness values, but it did not have a significant effect on other textural characteristics. L* values generally decreased, and b* values increased depending on çemen paste usage in hamburgers. Furthermore, a* and b* values of groups (CP8 and CP9) containing high amounts of SRP and GP in çemen paste were generally found to be higher than in control or BC. The lower TBARS and LPO levels were generally determined in hamburgers (CP3, CP6, and CP9) containing a low amount of FSF and a high amount of GP in çemen paste. Moreover, çemen paste generally did not exhibit significant antimicrobial activity. Based on the above results, it can be stated that çemen paste has the potential to improve quality characteristics and delay oxidative changes in hamburgers. Especially, çemen paste formulation used in CP9 (2% FSF, 3.5% SRP, and 4.5% GP) can be recommended in hamburger production for retarding the lipid oxidation.

## AUTHOR CONTRIBUTIONS

**Ömer Köker:** Data curation (equal); formal analysis (equal); investigation (equal). **Birol Kılıç:** Conceptualization (equal); methodology (equal); supervision (lead); writing – review and editing (lead). **Azim ŞİmŞek:** Conceptualization (equal); data curation (equal); formal analysis (equal); methodology (equal); writing – original draft (equal).

## CONFLICT OF INTEREST STATEMENT

Authors do not have any conflict of interest to disclose nor do they endorse the use of any product/technology/service over the other.

## ETHICS STATEMENT

This research does not require ethics approval.

## Data Availability

The data used to support the findings of this study are available from the corresponding author upon reasonable request.
